# Vasorelaxant and Blood Pressure-Lowering Effects of *Cnidium monnieri* Fruit Ethanol Extract in Sprague Dawley and Spontaneously Hypertensive Rats

**DOI:** 10.3390/ijms25084223

**Published:** 2024-04-11

**Authors:** Junkyu Park, Sujin Shin, Youngmin Bu, Ho-young Choi, Kyungjin Lee

**Affiliations:** 1 Department of Science in Korean Medicine, Graduate School, Kyung Hee University, Seoul 02447, Republic of Korea; ojeoksan@khu.ac.kr; 2 Department of Korean Medicine, Graduate School, Kyung Hee University, Seoul 02447, Republic of Korea; sjshin04@khu.ac.kr; 3 Department of Herbal Pharmacology, College of Korean Medicine, Kyung Hee University, Seoul 02447, Republic of Korea; ymbu@khu.ac.kr (Y.B.); hychoi@khu.ac.kr (H.-y.C.)

**Keywords:** *Cnidium monnieri* fruit, hypertension, vasorelaxation, osthole, imperatorin

## Abstract

*Cnidium monnieri* (L.) Cusson, a member of the *Apiaceae* family, is rich in coumarins, such as imperatorin and osthole. *Cnidium monnieri* fruit (CM) has a broad range of therapeutic potential that can be used in anti-bacterial, anti-cancer, and sexual dysfunction treatments. However, its efficacy in lowering blood pressure through vasodilation remains unknown. This study aimed to assess the potential therapeutic effect of CM 50% ethanol extract (CME) on hypertension and the mechanism of its vasorelaxant effect. CME (1–30 µg/mL) showed a concentration-dependent vasorelaxation on constricted aortic rings in Sprague Dawley rats induced by phenylephrine via an endothelium-independent mechanism. The vasorelaxant effect of CME was inhibited by blockers of voltage-dependent and Ca^2+^-activated K^+^ channels. Additionally, CME inhibited the vascular contraction induced by angiotensin II and CaCl_2_. The main active compounds of CM, i.e., imperatorin (3–300 µM) and osthole (1–100 µM), showed a concentration-dependent vasorelaxation effect, with half-maximal effective concentration values of 9.14 ± 0.06 and 5.98 ± 0.06 µM, respectively. Orally administered CME significantly reduced the blood pressure of spontaneously hypertensive rats. Our research shows that CME is a promising treatment option for hypertension. However, further studies are required to fully elucidate its therapeutic potential.

## 1. Introduction

Hypertension, as a significant contributor to cardiovascular diseases, affected approximately 1.3 billion adults aged 30 to 79 worldwide in 2019. This prevalence reflects an almost twofold increase compared to figures from 1990 [1]. Mortality attributable to hypertension was estimated to be approximately 8.5 million individuals in 2015. Notably, 88% of this figure is disproportionately concentrated in low- and middle-income countries, where improvements in disease prevalence have been challenging to achieve [2].

Alterations in the equilibrium of the sympathetic nervous system, renin–angiotensin–aldosterone system, vascular tension, sodium levels, and cardiac function contribute to elevated peripheral vascular resistance and increased circulatory fluid volume, thereby fostering hypertension [3]. For hypertension prevention and treatment, a combination of antihypertensive agents (e.g., calcium channel blockers, thiazide diuretics, sympatholytic agents, or angiotensin receptor blockers) and non-pharmacological lifestyle modifications is recommended [4]. Despite the development of various treatments, 9–18% of patients with hypertension fail to achieve their target blood pressure with conventional drug therapy, a state defined as resistant hypertension [5]. Thus, there is a significant need for new and effective treatments for hypertension.

Currently, medicinal herbs are often considered as alternative treatments for hypertension and cardiovascular disease. These herbs contain various beneficial phytochemicals, and numerous scientific studies have investigated their effectiveness against cardiovascular diseases and hypertension [6]. For example, curcumin in *Curcuma longa* [7], tannins in *Sanguisorba officinalis* [8], and sakuranetin in cherry trees [9] are among the active components studied for their potential in the treatment of hypertension. Medicinal herbs have several advantages. They are generally less expensive than conventional treatments, considered safe, and may help overcome drug resistance [10,11]. According to an ethnobotanical study, the *Apiaceae* family accounts for one of the highest proportions of medicinal herbs that are effective against hypertension [12]. In previous studies, the roots of *Angelica decursiva* [13], *Ostericum Koreanum* [14], *Ligusticum jeholense* [15], *Angelica dahurica* [16], *Ligusticum wallichii* [17], *Angelica gigas* [17], *Bupleurum fruticosum* [18], and *Peucedanum japonicum* [19], along with the oil of *Ferula asafoetida* [20], *Trachyspermum ammi* [21], and the aerial part of *Heracleum sphondylium* [22], all belonging to the Apiaceae family, were investigated for their vasorelaxant effects and mechanisms. However, the vasorelaxation effect of *Cnidium monnieri* (L.) Cusson fruit (CM), a medicinal herb belong to the Apiaceae, has yet to be studied.

CM is widely distributed in China, Korea, Japan, and other regions and has been used to treat sexual dysfunction, such as male impotence, in traditional Eastern medicine [23]. CM and its major active components have been studied for their potential to treat male impotence by relaxing the peripheral vascular smooth muscles in the rabbit penile corpus cavernosum [24]. Peripheral vasodilation can act on the mechanism of reducing systemic vascular resistance and increasing blood flow, which may lead to lower blood pressure [25].

Experimental studies have provided evidence suggesting that CM may be effective across a wide range of conditions, including neuroprotective [26], anti-bacterial [27], anti-asthmatic [28], anti-cancer [29,30,31], anti-oxidative [32], anti-inflammatory [33], anti-diabetic [34], hormone regulation [35], and sexual dysfunction treatments [36]. However, there is insufficient research to determine whether CM can be helpful in treating cardiovascular diseases such as hypertension. The main active compounds in CM are coumarins such as osthole, bergapten, imperatorin, xanthotoxol, and columbianetin [23]. Coumarins are natural aromatic compounds, and their derivatives are believed to have potential therapeutic effects against hypertension [37]. Moreover, osthole and imperatorin relaxed the constricted rat aortic rings via the inhibition of the Ca^2+^ channel in a dose-dependent manner [38,39]. Additionally, imperatorin inhibited cardiac myocyte hypertrophy, which could attenuate hypertension, myocardial hypertrophy, and cardiac fibrosis [40]. Despite previous research, there is a lack of studies on the vasorelaxant and hypotensive effects of CM. Therefore, this study aimed to find out whether CM is potentially effective in treating hypertension and to explore its mechanisms of action. The vasorelaxant effect was evaluated by observing the thoracic aortic rings obtained from Sprague Dawley (SD) rats, and the acute hypotensive effect was evaluated using the tail cuff method in spontaneously hypertensive rats (SHRs).

## 2. Results

### 2.1. Qualitative and Quantitative High-Performance Liquid Chromatography (HPLC) Analysis of CM Extracts

**Figure 1:**
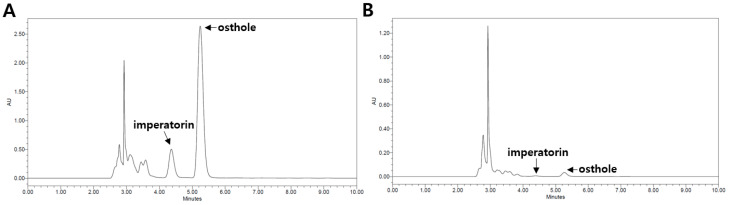
Qualitative and quantitative HPLC analysis of osthole and imperatorin in the fruit of *C. monnieri:* (**A**) 50% ethanol extract (CME); (**B**) water extract (CMW). The retention times of the peak levels of imperatorin and osthole were 4.37 min and 5.25 min, respectively.

The CM sample was reflux-extracted using distilled water (CMW) and 50% ethanol (CME). The contents of the major coumarin compounds, imperatorin and osthole, in the CME and CMW were determined using HPLC analysis. The retention times for imperatorin and osthole were 4.37 and 5.25 min, respectively. The regression equations for imperatorin and osthole were y = 10^7^x − 612,485 (0.9984) and y = 5 · 10^7^x − 5 · 10^6^ (0.9985), respectively, demonstrating good linearity. The contents of imperatorin and osthole in the CME were 6.38% and 7.23%, respectively. However, the contents of imperatorin and osthole in the CMW were 0.68% and 1.07%, respectively (Figure 1).

### 2.2. Vasorelaxant Effect of CME and CMW on Contractile Responses Induced by PE

**Figure 2:**
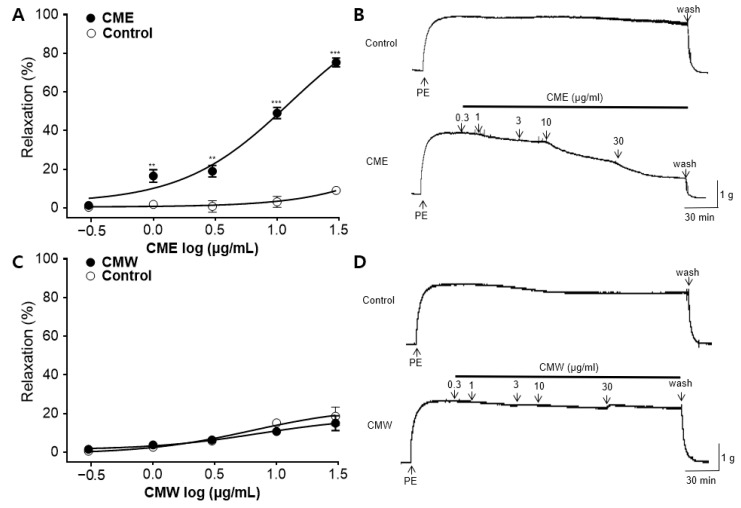
Vasorelaxant effects of *C. monnieri* 50% ethanol extract (CME) and water extract (CMW) on contractile responses induced by phenylephrine (PE): (**A**,**C**) Cumulative concentration–response curves and (**B**,**D**) representative traces of CME and CMW on endothelium-intact rat aortic rings pre-constricted with PE (1 μM). Unpaired *t*-test employed for statistical comparisons. Values are expressed as mean ± SEM (*n* = 4). ** *p* < 0.01 vs. control, *** *p* < 0.001 vs. control.

The vasorelaxant effects of the CME and CMW were evaluated using phenylephrine (PE) pre-constricted rings in Krebs–Henseleit (KH) buffer. The CME caused a relaxation response in a concentration-dependent manner (0.3, 1, 3, 10, and 30 μg/mL). The maximal vasorelaxant effect of the CME at 30 μg/mL on PE-induced contraction was 75.28 ± 2.28% (Figure 2A). The half-maximal effective concentration (EC_50_) of the CME was 10.19 ± 0.55 μg/mL. There was no significant difference between the CMW and the control until the dose reached 30 μg/mL (Figure 2C).

### 2.3. Vasorelaxant Effect of CME in Endothelium-Intact or Endothelium-Denuded Rat Aortic Rings

**Figure 3:**
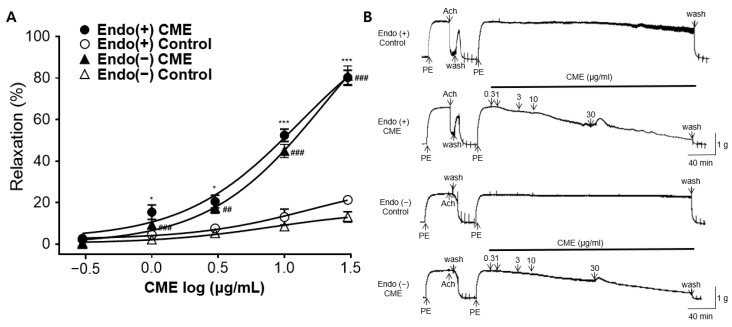
Vasorelaxant effects of *C. monnieri* 50% ethanol extract (CME) in endothelium-intact or endothelium-denuded rat aortic rings: (**A**) Cumulative concentration–response curves and (**B**) representative traces of CME on endothelium-intact [Endo (+)] or endothelium-denuded [Endo (−)] rat aortic rings pre-constricted with phenylephrine (PE, 1 μM). An unpaired *t*-test was employed for statistical comparisons. Values are expressed as mean ± SEM (*n* = 4). * *p* < 0.05, *** *p* < 0.001 vs. Endo(+) control. ## *p* < 0.01, ### *p* < 0.001 vs. Endo(−) control.

To assess whether the presence or absence of the endothelium was associated with vasorelaxation, the CME group was compared with the respective control groups. Regardless of the endothelium presence, the CME exhibited a concentration-dependent vasorelaxation effect. The maximal vasorelaxant effects of the CME in the endothelium-intact and endothelium-denuded rat aortic rings at 30 μg/mL were 80.26 ± 3.50% and 81.07 ± 4.78% (Figure 3A). There was no significant difference in the vasorelaxant effects based on the presence or absence of the endothelium.

### 2.4. Vasorelaxant Effect of CME on Aortic Rings Pre-Incubated with K^+^ Channel Blockers

**Figure 4:**
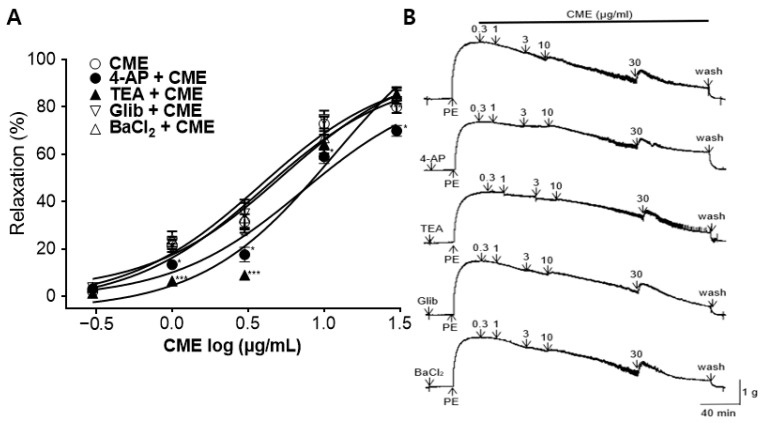
Effect of K^+^ channel blockers on *C. monnieri* 50% ethanol extract (CME)-induced vasorelaxation: (**A**) Cumulative concentration–response curves and (**B**) representative traces of rat aortic rings. K^+^ channel blockers included 4-aminopyridine (4-AP), tetraethylammonium (TEA), glibenclamide (Glib), and barium chloride (BaCl_2_), and the aortic rings were pre-constricted with phenylephrine (PE, 1 μM). Statistical comparisons were conducted using an unpaired *t*-test. Values are presented as mean ± SEM (*n* = 5). * *p* < 0.05, *** *p* < 0.001 vs. CME.

Pre-incubation with potassium channel blockers, such as 4-aminopyridine (4-AP), tetraethylammonium (TEA), glibenclamide (Glib), or Barium chloride (BaCl_2_), significantly decreased the CME-induced relaxation of the endothelium-intact aortic rings pre-constricted by PE. In the presence of 4-AP (1 mM), TEA (1 mM), Glib (10 µM), and BaCl_2_ (10 µM), the maximum relaxation effects of the CME were 69.93 ± 2.28%, 85.59 ± 2.81%, 82.30 ± 4.88%, and 84.78 ± 3.34%, respectively, at a 30 µg/mL concentration (Figure 4).

### 2.5. Effect of CME on Extracellular Ca^2+^-Induced Contraction Pre-Treated with PE and KCl

**Figure 5:**
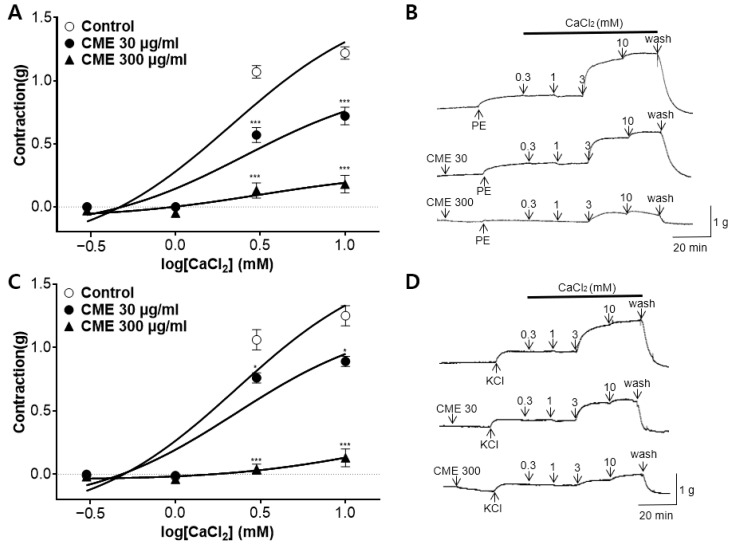
Effect of *C. monnieri* 50% ethanol extract (CME) on extracellular Ca^2+^-induced contraction pre-treated with phenylephrine (PE) and KCl. Inhibitory effect of CME (30 and 300 µg/mL) induced by extracellular CaCl_2_ (0.3–10 mM) constriction on aortic rings pre-treated with: (**A**) PE (1 μM), (**C**) KCl (60 mM), and (**B**,**D**) representative traces. Statistical comparisons were conducted using an unpaired *t*-test. Values are expressed as mean ± SEM (*n* = 4). * *p* < 0.05, *** *p* < 0.001 vs. control.

The cumulative addition of calcium chloride (CaCl_2_, 0.3–10 mM) gradually induced the contraction of the rat aortic rings pretreated with PE (1 µM) and potassium chloride (KCl, 60 mM) in the Ca^2+^-free KH buffer. Pre-treatment with CME (30 and 300 µg/mL) significantly attenuated the constriction induced by CaCl_2_ compared to that in the control group (Figure 5).

### 2.6. Effect of CME and SK&F96365 on PE-Induced Contraction in the Presence of Nifedipine

**Figure 6:**
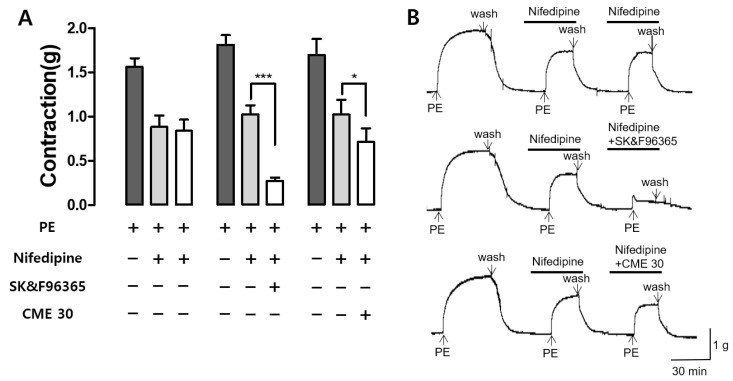
Effect of *C. monnieri* 50% ethanol extract (CME) and SK&F96365 on phenylephrine (PE)-induced contraction in the presence of nifedipine: (**A**) The effects and (**B**) traces of CME (30 µg/mL) on PE-induced contraction in the presence of nifedipine (10 µM). Statistical comparisons were conducted using an unpaired *t*-test. Values are expressed as mean ± SEM (*n* = 4). * *p* < 0.05, *** *p* < 0.001.

The effects of CME (30 µg/mL) on Ca^2+^-induced influx through receptor-operated Ca^2+^ channels (ROCCs) were confirmed by co-administration with the voltage-dependent Ca^2+^ channel (VDCC) blocker nifedipine. SK&F96365, an inhibitor of ROCCs, was utilized as a positive control. Nifedipine (10 µM) inhibited the second PE-induced contraction, and CME and SK & F9636 (50 µM) significantly reduced the third PE-induced contraction in the presence of nifedipine. Nifedipine blocked the VDCCs, SK&F96365 blocked the ROCCs, and the co-administration of nifedipine and SK&F96365 further reduced PE-induced contraction. Similarly, the CME pre-treatment reduced PE-induced contraction in the presence of nifedipine, suggesting that the CME inhibited the entry of extracellular Ca^2+^ channels via PE-activated ROCCs (Figure 6).

### 2.7. Inhibitory Effect of CME Pre-Treatment on Ang II-Induced Contraction

**Figure 7:**
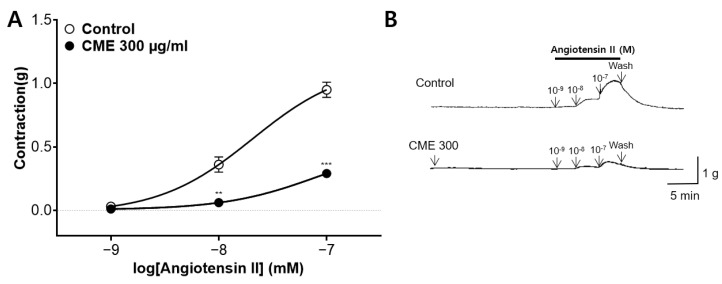
Inhibitory effects of *C. monnieri* 50% ethanol extract (CME) pre-treatment on angiotenin II (Ang II)-induced contraction. (**A**) Inhibitory effect and (**B**) representative original traces of CME (300 µg/mL) are shown in the contraction induced by Ang II (10^−9^–10^−7^ M) on the endothelium-intact aortic rings. Statistical comparisons were conducted using an unpaired *t*-test. Values are expressed as the mean ± SEM (*n* = 4). ** *p* < 0.01, *** *p* < 0.001 vs. control.

We assessed the inhibitory effect of CME (300 µg/mL) on angiotenin II (Ang II, 10^−9^–10^−7^ M)-induced vasoconstriction of the endothelium-intact aortic rings. The CME pre-treatments significantly inhibited the constrictions to 0.29 ± 0.03 (g), while the constriction levels in the control group were 0.95 ± 0.06 (g) (Figure 7).

### 2.8. Vasorelaxant Effect of the Main Active Components in CME, Osthole, and Imperatorin

**Figure 8:**
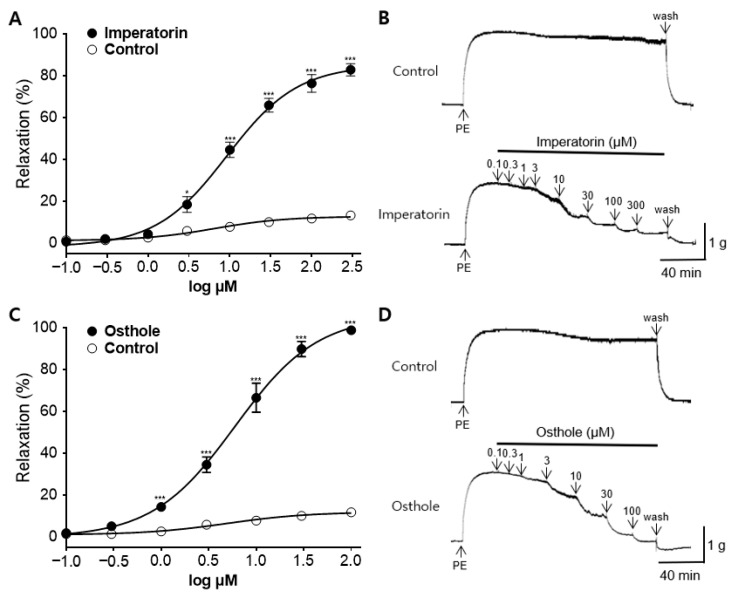
Vasorelaxant effect of the main active components in *C. monnieri* 50% ethanol extract (CME): (**A**,**C**) Cumulative concentration–response curves and (**B**,**D**) representative traces of imperatorin and osthole on endothelium-intact rat aortic rings pre-constricted with phenylephrine (PE, 1 μM). Statistical comparisons were conducted using an unpaired *t*-test. Values are expressed as mean ± SEM (*n* = 4). * *p* < 0.05, *** *p* < 0.001 vs. control.

The vasorelaxant effects, minimum effective doses, and EC_50_ values of imperatorin and osthole, the main active components of CM, were investigated in the endothelium-intact aortic rings. Imperatorin exhibited dose-dependent vasorelaxant activity from a concentration of 3 µM, with a maximal vasorelaxant effect of 82.77 ± 2.93% at 300 µM. Osthole, on the other hand, showed dose-dependent vasorelaxant activity from a concentration of 1 µM, with a maximal vasorelaxant effect of 98.71 ± 1.09% at 100 µM. The EC_50_ values of imperatorin and osthole were 9.14 ± 0.06 and 5.98 ± 0.06 µM, respectively (Figure 8).

### 2.9. Hypotensive Effect of CME in SHR

To investigate the hypotensive effects of CME, systolic blood pressure (SBP) and diastolic blood pressure (DBP) were measured at the baseline and 1, 2, 4, 8, and 12 h after administering 100, 300, or 1000 mg/kg of CME to the SHRs. The SBP and DBP values of the CME-treated groups were compared with those of the control group. The positive control group was intraperitoneally administered with 1 mg/kg amlodipine and compared with the vehicle control group.

**Figure 9:**
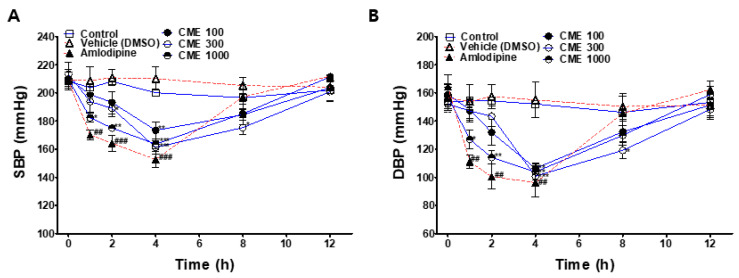
Hypotensive effect of *C. monnieri* 50% ethanol extract (CME) in spontaneously hypertensive rats. Systolic blood pressure (**A**) and diastolic blood pressure (**B**) were measured at the baseline and 1, 2, 4, 8, and 12 h after CME (100, 300, and 1000 mg/kg, p.o.) administration. Amlodipine (1 mg/kg, i.p.) was used as a positive control. Vehicle group treated with dimethyl sulfoxide (DMSO, i.p.) in the same capacity as a solvent. Values are expressed as the mean ± SEM (*n* = 4). Two-way ANOVA and an unpaired *t*-test were used for statistical comparisons. * *p* < 0.05, ** *p* < 0.01, *** *p* < 0.001 vs. control. ## *p* < 0.01, ### *p* < 0.001 vs. vehicle (DMSO).

The SBP and DBP values of all CME-administered groups significantly decreased 4 h after administration and returned to the baseline at 12 h after administration. The group administered with 1000 mg/kg CME showed a significant hypotensive effect from 1 h to 4 h after administration. At 4 h after the administration of 1000 mg/kg of CME, the SBP was lowered from 209.6 ± 5 to 163.6 ± 3.5 mmHg, and the DBP decreased from 157.2 ± 9.6 to 103.8 ± 5.9 mmHg (Figure 9).

## 3. Discussion

CM was extracted using two solvents: water and 50% ethanol. According to the study results, the 50% ethanol extracts of CM demonstrated concentration-dependent vasorelaxant effects on the thoracic aorta of SD rats, while the water extracts showed no significant effects. These findings suggest that the use of organic solvents, such as ethanol, is advantageous for extracting active ingredients that induce vasodilation in CM. CME exhibited a maximum relaxation effect of 75.28 ± 2.28% at 30 μg/mL, and the EC_50_ value of the CME was 10.19 ± 0.55 μg/mL. Subsequent investigations into the vasorelaxant mechanisms and antihypertensive effects of CME were performed.

Vasorelaxant effects are mediated by endothelium-dependent and endothelium-independent mechanisms. The endothelium-dependent mechanisms are related to the actions of the nitric oxide (NO)/soluble guanylate cyclase (sGC)/cyclic guanosine-3′,5′-monophosphate (cGMP) [41], and prostacyclin (PGI_2_) pathways [42], and the endothelium-independent mechanisms are associated with the action of vascular smooth muscle cells (VSMCs) [43]. Mechanism studies were conducted to determine whether the action of the CME was endothelium-dependent or associated with K^+^ channels, Ca^2+^ channels, and angiotensin receptors.

The endothelium plays a vital role in regulating vascular tone. NO, PGI_2_, and endothelium-derived hyperpolarization factor (EDHF) are released from the endothelium, inducing vasorelaxation of the rat thoracic aorta [44]. NO is produced through the conversion of L-arginine by nitric oxide synthase (NOS). NO then activates sGC, resulting in an elevation of cGMP. Finally, this process leads to calcium depletion from cytosolic space and relaxation of the vascular smooth muscle [41]. The vasorelaxant effects of CME showed no significant differences based on the presence or absence of the endothelium, indicating no association with the NO/sGC/cGMP pathway. Therefore, additional experiments using L-NAME (NOS inhibitor), ODQ (sGC inhibitor), or MB (cGMP inhibitor) were not performed.

K^+^ channels play a crucial role in regulating VSMC contraction and growth. As the predominant ion conductor of VSMCs, they play a vital role in determining and regulating the membrane potential [45]. To study the vasorelaxant effect of CME on K^+^ channels, 4-AP (a voltage-dependent K^+^ channel blocker), TEA (a Ca^2+^-activated K^+^ channel blocker), Glib (an ATP-sensitive K^+^ channel blocker), or BaCl_2_ (an inwardly rectifying K^+^ channel blocker) were treated before the PE-induced vasoconstriction. 4-AP pre-treatment significantly inhibited the vasorelaxant effects of CME. The TEA pre-treatment also inhibited the vascular relaxation effect of CME at low concentrations (1–3 μg/mL). However, there were no significant differences after pretreatment with Glib or BaCl_2_. These results indicate that the vascular relaxation effect of CME is associated with voltage-dependent K^+^ channels (K_V_). The vasorelaxant effect of CME appears to be partly related to Ca^2+^-activated K^+^ channels (K_Ca_).

In addition to K^+^ channels, Ca^2+^ channels also play a key role in vascular smooth muscle contraction. Vasoconstrictors elevate the concentration of free calcium in cytosol, whereas vasodilators either decrease calcium levels or prevent vasoconstrictor-induced increases [46]. Various Ca^2+^-elevating pathways regulate the contractility of vascular smooth muscles in isolated rat aortic rings, receptor-operated Ca^2+^ channels (ROCCs), voltage-dependent Ca^2+^ channels (VDCCs), and intracellular Ca^2+^ release [47]. The sarcoplasmic reticulum initiates the release of Ca^2+^ stored within the cell, which is triggered by the production of inositol 1,4,5-trisphosphate (IP_3_) in cytosol. As a result, vascular smooth muscles develop tension with a much shorter latency through the ROCC and VDCC pathways, which are activated by extracellular Ca^2+^ influx [46]. To investigate the mechanism of action of Ca^2+^ channels, rat thoracic aortas were contracted using PE and KCl. The vasoconstriction mechanism of PE involves the activation of VDCCs and ROCCs [48]. In contrast to PE, KCl circumvents ROCCs and activates VDCCs. Pre-incubation with CME (30 and 300 μg/mL) affected vasoconstriction induced by PE or KCl after Ca^2+^ supplementation in Ca^2+^ -free KH buffer. These results show that the vascular relaxation effect of CME is related to extracellular Ca^2+^ through ROCCs and VDCCs, and that this mechanism is similar to that of osthole and imperatorin, the main components of CM [39,49]. Additionally, we investigated whether CME inhibits contraction mediated by ROCCs using nifedipine, a VDCC and intracellular Ca^2+^ release blocker. SK&F96365, a selective ROCC inhibitor, was used as the positive control. The results revealed that, in the presence of nifedipine blocking VDCCs, CME (30 μg/mL) further inhibited contraction, similar to SK&F 96365. These results suggest that CME inhibits the entry of extracellular Ca^2+^ via ROCCs, activated by PE.

Ang II is a pivotal factor in the pathophysiology of cardiovascular diseases and serves as the main effector of the renin–angiotensin system [50]. The action of renin produced in the liver leads to the production of Ang II, which causes the smooth muscle walls of the arterioles to contract, thereby increasing blood pressure. It also stimulates aldosterone secretion from the adrenal glands and vasopressin secretion from the hypothalamus. Aldosterone and vasopressin retain sodium in the kidneys, reducing water excretion and increasing blood volume and pressure [51]. To investigate whether CME affects Ang II, the thoracic aorta of rats was pretreated with CME (300 μg/mL), followed by the cumulative administration of Ang II (10^−9^–10^−7^). CME significantly reduced the contraction induced by Ang II, implying that CME hindered the binding of Ang II to its receptor.

In previous studies, CM was believed to be effective against hypertension due to the action of imperatorin and osthole. The contents and effective dosages of imperatorin and osthole were measured to determine whether they play major roles in the vasorelaxant effects of CME. Based on HPLC analysis of 30 µg/mL CME, the imperatorin and osthole contents were 8.02 µM and 7.84 µM, respectively. Considering that the EC_50_ values for imperatorin and osthole were 9.14 ± 0.06 and 5.98 ± 0.06 µM, respectively, it can be inferred that these two components play a significant role in inducing the vascular relaxation effect of CME. The vasorelaxant mechanisms of imperatorin and osthole, as revealed in previous studies, are similar to those of CME. These mechanisms are endothelium-dependent and involve the blocking of Ca^2+^ channels (ROCCs and VDCCs) and the renin–angiotensin system [40,49,52,53]. Imperatorin induces vasorelaxation through K_Ca_ and K_V_-related mechanisms, with an association with the ATP-sensitive K^+^ channel observed solely at concentrations above 100 μM In contrast, osthole is not associated with K_V_-related mechanisms [54], and there has been no research on K_Ca_-related mechanisms. The K^+^ channel-mediated mechanism of CME is presumed to involve the action of imperatorin. However, since only a few active compounds of vasorelaxation mechanisms are known, and the amount of each component in the extract has not been quantified, there is a limitation in estimating the main active compound; therefore, further research is needed.

To confirm the hypotensive action of CME, the SBP and DBP of the SHRs were measured after oral administration of the drug using a non-invasive tail-cuff method. The SBP and DBP significantly decreased after 4 h of CME administration (100, 300, and 1000 mg/kg), suggesting that it may be effective in lowering blood pressure in humans when consumed at doses higher than 8.11 mg/kg. As a positive control, amlodipine (1 mg/kg) was injected intraperitoneally, and both the SBP and DBP significantly decreased at 1, 2, and 4 h after injection. Twelve hours after CME administration, the blood pressure recovered to the baseline.

In this study, because the hypotensive effect of CME was confirmed only after a single administration, it is necessary to confirm the effectiveness and safety of long-term repetitive administration. Imperatorin and osthole, the main active ingredients of CME, act by inhibition of the L-type calcium channels [40,54]. As they act on the same pathway as L-type calcium channel blockers, such as nifedipine and verapamil, there is a risk of an antagonizing effect or overactivation of the conventional drug function when used simultaneously; therefore, care should be taken. Further research is necessary to investigate the drug–drug interactions between CME and conventional medications.

## 4. Materials and Methods

### 4.1. Materials and Chemicals

BaCl_2_, glucose, magnesium sulfate (MgSO_4_), potassium phosphate monobasic (KH_2_PO_4_), KCl, sodium chloride (NaCl), sodium hydrogen carbonate (NaHCO_3_), CaCl_2_, and urethane were sourced from Daejung Chemicals & Metals Co., Ltd. (Siheung, Republic of Korea). Dimethyl sulfoxide (DMSO) was purchased from Junsei (Tokyo, Japan). Phenylephrine, acetylcholine (Ach), and ethylene glycol-bis(2-aminoethylether)-N,N‚N′,N′-tetraacetic acid (EGTA), Ang II, SK&F96365, nifedipine, imperatorin (>98.0% purity verified by HPLC, CAS: 482-44-0), and osthole (≥95.0% purity verified by HPLC, CAS: 484-12-8) were purchased from Sigma Aldrich, Inc. (St. Louis, MO, USA). 4-AP, Glib, and TEA were obtained from Wako Pure Chemical Industries, Ltd. (Osaka, Japan).

### 4.2. Sample Preparation

**Figure 10:**
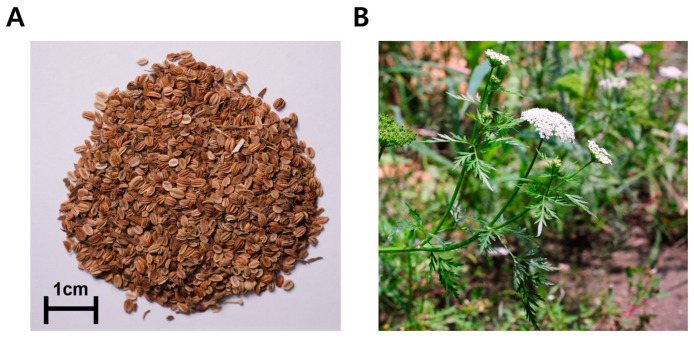
The morphology of *Cnidium monnieri* (L.) Cusson used in the experiment: (**A**) the fruit; (**B**) the whole plant. The photographs were taken before the experiment.

CM was purchased from Omniherb Co. (Daegu, Republic of Korea) in February 2023 and originated from China. The morphologies of the samples used in the experiments are shown in Figure 10. The dried products were processed into a powder using a blender, and 40 g of the powder was extracted with 400 mL of distilled water at 97 ± 3 °C and 400 mL of 50% ethanol at 75 ± 3 °C for 2 h, respectively. Each solution was vacuum-filtered using qualitative filter paper (HM, No.2, Seoul, Republic of Korea), and the concentrates were freeze-dried. The yields of CME and CMW are presented in Table 1. The extracted powder was stored at −20 °C.

### 4.3. HPLC Analysis of CME and CMW

Accurately weighed amounts of CME and CMW (10 mg) were dissolved in 1 mL of methanol (HPLC grade, J. T. Baker Co. Ltd., New Jersey, NJ, USA). The extract was then filtered twice through a 0.45 μm syringe filter (PVDF, Korea Ace Science, Republic of Korea). Imperatorin and osthole were used as standards for the qualitative analysis of CME and CMW. These standards were serially diluted (0.0625, 0.125, 0.25, 0.5, and 1 mg/mL), and HPLC chromatograms were obtained. The relationship between the concentration and the peak area was determined using the least-squares method (R^2^ value). HPLC analysis was conducted using a Waters e2695 Alliance HPLC system connected to a PDA Detector 2998, and Empower2 Software was used for analysis. A YMC-Triart 4.60 × 250 mm C_18_ reversed-phase column with 5-μm particles was utilized.

The mobile phase consisted of acetonitrile and water (HPLC grade, J. T. Baker Co., Ltd., New Jersey, NJ, USA) in an 80:20 (*v*/*v*) ratio. Chromatography was performed at room temperature with a flow rate of 2.0 mL/min, and 10 μL was analyzed for 10 min. The column eluent was monitored at 320 nm, and all solvents were degassed using a micromembrane filter.

### 4.4. Animals

A total of 31 healthy male SD rats aged 6–8 weeks and weighing 200–250 g (Daehan Biolink, Eumseong-gun, Republic of Korea) were used for the ex vivo rat aortic ring assay. A total of 24 male SHRs aged 10 weeks and weighing 280–300 g (SLC, Inc., Shizuoka, Japan) were used for the blood pressure measurements. All rats were housed in a controlled environment at 22 ± 2 °C and had access to tap water and food ad libitum. The experimental procedures adhered to the Animal Welfare Guidelines of the Animal Experiment Ethics Committee of Kyung Hee University and were approved by the committee (KHSASP-23-506).

### 4.5. Confirmation of the Mechanism Associated with Vasodilatory Effect

#### 4.5.1. Preparation of Rat Aortic Rings

The animals were anesthetized through an intraperitoneal injection (i.p.) of urethane at a dose of 1.2 g/kg body weight. The thoracic aorta was dissected, and the isolated aorta was transferred to a Petri dish containing KH buffer (NaCl, 118.0 mM; KCl, 4.7 mM; CaCl_2_, 2.5 mM; MgSO_4_, 1.2 mM; KH_2_PO_4_, 1.2 mM; and NaHCO_3_, 25.0 mM). After removing the connective tissue and fat attached to the blood vessels, the thoracic aorta was divided into 2–3 mm long pieces. Each piece was suspended between two tungsten stirrups in an organ bath containing 10 mL of KH buffer and maintained at 37 °C with a continuous supply of a mixed gas composed of 95% O_2_ and 5% CO_2_. The aortic ring pieces were equilibrated at 1.0 g tension for 40 min. The KH buffer was refreshed every 10 min during the equilibration period.

#### 4.5.2. Measurement of Tension in Rat Aortic Rings during PE-Induced Constriction

To assess the effect of samples on vasorelaxation, the constriction of aortic rigs was conducted using PE at a concentration of 1 μM following an equilibration period. Once the tension reached a plateau, the samples were introduced into the organ chamber at incrementally increasing concentrations.

#### 4.5.3. Role of CME on Endothelium-Intact and Endothelium-Denuded Rat Aortic Rings

To verify the potential involvement of endothelial cells in vasodilation induced by CME, the vasorelaxant effects of CME were assessed, regardless of the vascular endothelium. To confirm whether the endothelium was intact or denuded, the rat aorta rings were pre-constricted with PE (1 μM) and then relaxed with Ach (10 μM). The aortic rings that exhibited more than 80% relaxation in response to Ach were categorized as endothelium-intact, whereas those with less than 10% relaxation were considered endothelium-denuded. After confirmation, the aortic rings were thoroughly washed with KH buffer.

#### 4.5.4. Role of K^+^ Channel on the Effect of CME

To elucidate the underlying mechanisms involving K^+^ channels, the endothelium-intact rings were pre-incubated with specific blockers: 4-AP (K_V_ blocker, 1 mM), TEA (K_Ca_ blocker, 1 mM), Glib (non-specific ATP-sensitive K^+^ channel blocker, 10 μM), or BaCl_2_ (inwardly rectifying K^+^ channel blocker, 10 μM). The incubation period was 20 min prior to the pre-constriction induced by PE.

#### 4.5.5. Role of CME on Extracellular Ca^2+^-Induced Contraction

To investigate the underlying mechanisms of Ca^2+^ channels, the rat aorta rings were incubated in Ca^2+^-free KH buffer containing ethylene glycol-bis(2-aminoethylether)-N,N,N′,N′-tetraacetic acid (EGTA) at a concentration of 1 mM. A pre-treatment of CME (30 and 300 μg/mL) was applied to the aortic rings for 20 min. Subsequently, PE (1 μM) or KCl (60 mM) was administered to activate the ROCCs or VDCCs. Following this, CaCl_2_ was cumulatively added at concentrations ranging from 0.3 to 10.0 mM to assess the effect of CME on the vasoconstriction induced by Ca^2+^ channels.

#### 4.5.6. Role of CME in PE-Induced Contraction in the Presence of Nifedipine

To investigate the impact of CME on Ca^2+^ influx through ROCCs, we assessed the effects of CME (30 μg/mL), along with the ROCC blocker SK & F 9636 (50 μM), on nifedipine (1 μM)-induced contraction in the presence of voltage-dependent calcium channels. PE was administered twice in the presence of nifedipine, and the aortic rings were treated with CME or SK&F 96365 before the second application of PE.

#### 4.5.7. Role of CME Pre-Treatment on Ang II-Induced Contraction

To explore the vasorelaxant mechanism associated with the angiotensin receptor, the aortic rings were pre-incubated with CME (10 μg/mL) for 20 min. Subsequently, Ang II was cumulatively administered in concentrations ranging from 10^−9^ to 10^−7^ M. This assay was performed to measure the inhibitory effects of CME on Ang II-induced vasoconstriction.

### 4.6. Measurement of Blood Pressure

The SBP and DBP were measured in the SHRs using the non-invasive tail-cuff method with the CODA 8-Channel High Throughput Noninvasive Blood Pressure System (Kent Scientific Co., Ltd., Torrington, CT, USA). The animals were placed in a restraint apparatus equipped with an adjustable nose cone holder to restrict excessive movement and a rear gate providing free access to the base of the animal’s tail. The SBP and DBP of the SHRs were measured and recorded using an occlusion cuff and volume pressure recording (VPR) sensor.

To validate the hypotensive effects of the samples, 24 animals were randomly divided into 6 groups. Each group received oral doses of the samples (100, 300, and 1000 mg/kg), whereas the control group received distilled water. The positive control group was intraperitoneally administered amlodipine (1 mg/kg) dissolved in DMSO, and the vehicle group was administered DMSO at the same volume.

The blood pressure of the SHRs was measured before drug administration and at 1, 2, 4, 8, and 12 h after drug administration. Throughout the experiment, the surface temperature of the animals was maintained at 32–35 °C using a heating pad.

### 4.7. Data Analysis

The values are presented as the mean ± standard error of the mean (SEM). Statistical analyses were performed using GraphPad Prism 5 software (San Diego, CA, USA). Two-way analysis of variance (ANOVA) and unpaired *t*-tests were used for statistical comparisons. Statistical significance was set at *p* < 0.05.

## 5. Conclusions

This study confirmed that CME induces the endothelium-independent relaxation of isolated rat aortic rings via the regulation of VSMCs and is effective in treating hypertension. Considering the ingredient content and effective dosage as well as the mechanism of vasorelaxation, it is presumed that imperatorin and osthole are the main components responsible for the blood pressure-lowering effect of CME. However, further research is required to develop CME as a new drug or functional food supplement for the treatment of hypertension. It would be beneficial to explore whether CME is effective in other animal models of cardiovascular diseases, such as the pulmonary hypertension model, Ang II model, or L-NAME model. Additionally, further evidence regarding its safety, stability, and efficacy will need to be established to advance toward clinical trials.

## Figures and Tables

**Table 1:** Extracts used in this study.

Plant and Part	Solvent	Yield (%)	Abbreviation	Region
*Cnidium monnieri* (L.) Cusson Fruit	Water	8.05	CMW	China
*Cnidium monnieri* (L.) Cusson Fruit	50% Ethanol	8.10	CME	China

## Data Availability

The data presented in this study are available from the corresponding author upon request.
